# Estimation of the Volatility and Apparent Activity Coefficient of Levoglucosan in Wood-Burning Organic Aerosols

**DOI:** 10.1021/acs.estlett.4c00608

**Published:** 2024-11-03

**Authors:** Jun Zhang, Andreas Zuend, Jens Top, Mihnea Surdu, Imad EI Haddad, Jay G. Slowik, Andre S. H. Prevot, David M. Bell

**Affiliations:** † PSI Center for Energy and Environmental Sciences, Paul Scherrer Institute (PSI), 5232 Villigen, Switzerland; ‡ Department of Atmospheric and Oceanic Sciences, McGill University, Montréal, Quebec H3A 0B9, Canada

**Keywords:** levoglucosan, volatility, activity coefficient, wood burning

## Abstract

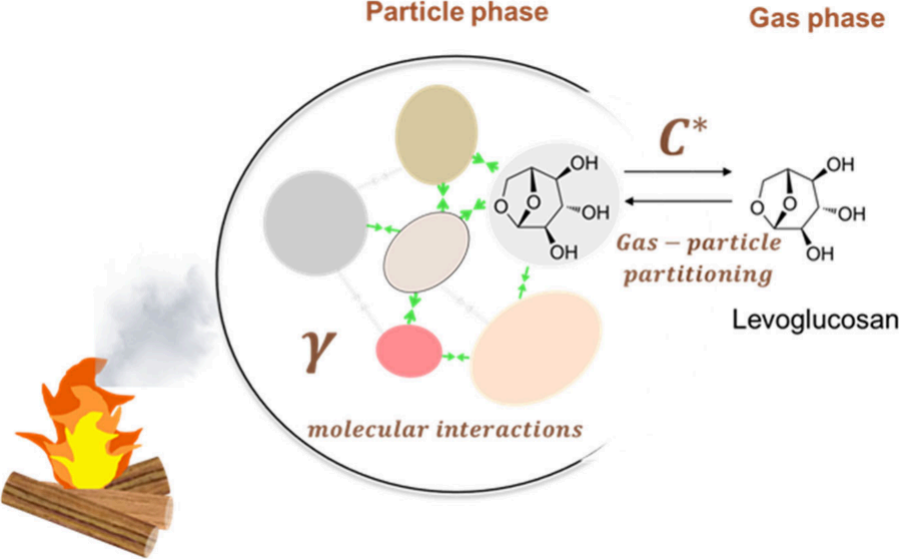

Biomass burning (BB) is a major source of aerosols and black carbon, thereby exerting an important impact on climate and air quality. Levoglucosan is the most well-recognized organic marker compound of BB and has been used to quantitatively assess BB’s contribution to ambient aerosols. However, little is known about levoglucosan’s evaporation under atmospheric conditions, primarily due to the uncertainty of its effective saturation vapor concentration (C*) and its unknown activity coefficient (γ), in the complex BB emission matrix. Here, we utilized a thermodenuder to investigate the evaporation of levoglucosan from mixtures with polyethylene glycol (PEG) or BB primary organic aerosol (BBPOA) matrices, respectively. We estimate a pure component log_10_(C*/[μg m^–3^]) of levoglucosan of 1.1 ± 0.1 at 298 K. We reveal that levoglucosan mixed with PEG or BBPOA becomes more volatile than when treated as a single component due to nonideal molecular interactions. Considering that phase separation might occur in such systems, we term γ apparent activity coefficient (γ_*a*_). We estimate log_10_C* and γ_*a*_ of levoglucosan in BBPOA of 1.8 ± 0.1 and 3.8 ± 0.3, assuming a liquid phase state. Consequently, γ_*a*_ must be considered to avoid significant underestimation of levoglucosan evaporation via gas–particle partitioning during transport.

## Introduction

Biomass burning (BB) is a globally occurring phenomenon and a major emission source of primary organic aerosol (POA) and organic gases.^1,2^ These emissions play an important role in air quality, human health, and climate.^3,4^ Levoglucosan (1,6-anhydro-β-d-glucopyranose), formed via the pyrolysis of cellulose and hemicellulose, has been identified as the most abundant compound in biomass-burning POA (BBPOA)^5^ and consequently is widely used as a molecular tracer for BB events.^6,7^ Nevertheless, levoglucosan is semivolatile,^8^ evaporating during transport due to air parcel dilution and temperature changes, and subsequently reacting with hydroxy radicals (OH) in the gas phase^8^ or undergoing heterogeneous oxidation by OH,^9^ which introduces uncertainty for the estimation of BBPOA contribution to aerosols. Therefore, accurately estimating the volatility of levoglucosan in ambient aerosols is crucial for determining its atmospheric lifetime.

The volatility of a compound in its pure form can be described by the liquid-state saturation vapor pressure (*p*^0^) or equivalently saturation concentration (*C*^0^) at a certain temperature and pressure. The *C*^0^ of levoglucosan has been experimentally determined.^8,10−12^ However, for multicomponent ambient aerosols, deviations caused by nonideal molecular interactions must be considered when deriving the effective saturation vapor concentration (C*) of levoglucosan from Raoult’s law. Therefore, the activity coefficient (γ), which corrects for the influence of nonideal heteromolecular interactions, is of great importance for obtaining C*.^13,14^ In atmospherically relevant situations, both positive and negative deviations from Raoult’s law have been observed. Laboratory studies have shown significant variability in the activity coefficients of the individual components in dicarboxylic acid mixtures. Lower-molecular-weight diacids have activity coefficients below 1, while heavier diacids have activity coefficients greater than 1.^15,16^ Recent research found that when α-pinene/O_3_ secondary organic aerosol (SOA) condenses on different seed particles, the apparent activity coefficient of the bulk SOA ranged from 1 to 5, primarily due to differences in polarity between the SOA and the seed particles.^17^ Unfortunately, in the real atmosphere, the incorporation of thousands of unknown compounds into activity coefficient models poses a significant challenge, and experimental data of activity coefficients in organic mixtures are elusive. As a result, few studies have reported the C*of levoglucosan, and none has considered its dependence on the BBPOA matrix.

In this study, we present the C* of levoglucosan in BBPOA and a novel method to obtain γ. A thermodenuder (TD) is deployed to investigate the evaporation behavior of levoglucosan in 3 scenarios: pure levoglucosan at 30% RH, levoglucosan in polyethylene glycol (PEG) solution at 30% RH, and levoglucosan emitted in BBPOA at 50% RH. The quantity of particle-phase levoglucosan in different mixtures is measured by an online extractive electrospray ionization time-of-flight mass spectrometer (EESI-TOF) as a function of TD temperature. The C* is computed based on thermograms of levoglucosan with an evaporation model, and the γ is retrieved by comparing it with *C*^0^ from the pure substance. This paper highlights the aerosol matrix effect on levoglucosan’s volatility and provides a new method for a measurement-derived determination of γ of ambient-relevant species.

## Methods and Materials

The experimental setup is shown in Figure S1. The BBPOA was generated by burning spruce logs in a stove. The combustion emissions were first introduced into a holding tank (1 m^3^, stainless steel) with an ejection diluter (DI-1000, Dekati Ltd.), where they were stored as the source of POA during evaporation experiments. When the mass concentration reached ∼50 μg/m^3^ after ∼30 dilutions in the sampling lines, the injection was stopped. The emissions in the sampling line from the holding tank were held at ∼50% RH by adding humidified air. The particles were then size-selected with an aerodynamic aerosol classifier (AAC, Cambustion). Gases from the emissions were removed by a charcoal denuder, and only particles entered the TD (Aerodyne),^18^ where volatile components evaporate with increasing temperature. Evaporating species were removed with another charcoal denuder to avoid condensation. The particle mass and chemical composition were monitored by a scanning mobility particle sizer (SMPS, TSI model 3938) and EESI-TOF (Tofwerk), respectively. The EESI-TOF signal responds linearly to the water-soluble organic mass,^19,20^ which is demonstrated in Figure S2.

Alternatively, upstream of the AAC, aerosols of pure levoglucosan (99%, Aldrich) or a mixture of levoglucosan and PEG (polyethylene glycol with a molecular weight of 300, Aldrich) were generated separately with a nebulizer. The dry mass fractions of levoglucosan in aqueous solution with PEG were 10%, 20%, and 50% for investigations of the matrix effect. After drying (to RH ∼ 30%), the particles were passed through the AAC and the TD as in the BBPOA experiments.

Characterization of the TD and determination of C* and γ of levoglucosan are shown in the SI.

## Results and Discussion

**Figure 1:**
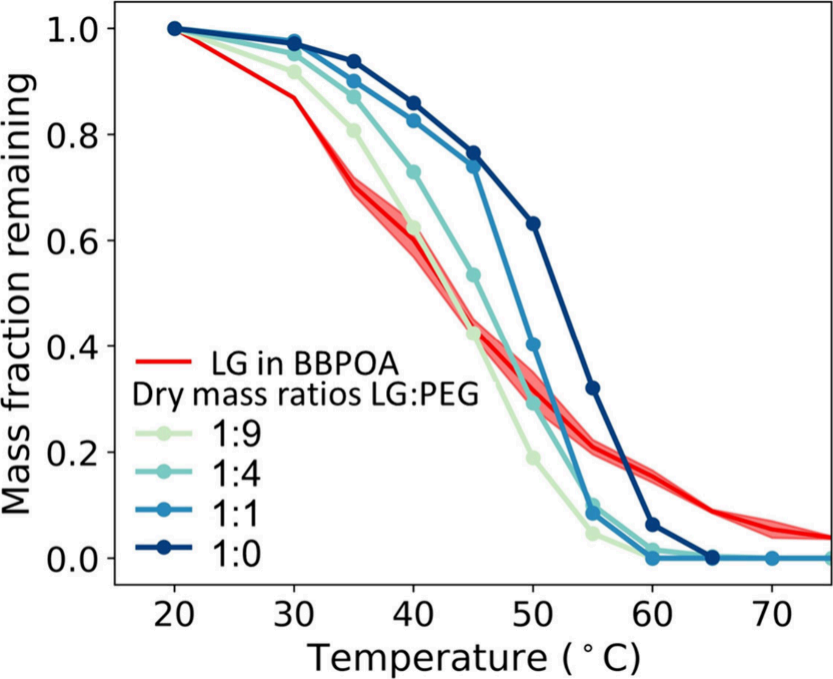
Thermogram measurement of levoglucosan (LG) in different matrices with a particle aerodynamic diameter at 300 nm.

Figure 1 presents the evaporation dynamics of levoglucosan in spruce-burning aerosols and in particles composed of levoglucosan and PEG, featuring dry mass ratios of levoglucosan to PEG of 10%, 20%, 50%, and 100%, meaning the water content is not included. For the particles of levoglucosan and PEG at 300 nm aerodynamic diameter at 30% RH, the mass fraction remaining of levoglucosan decreased due to evaporation with increasing temperature, and little remains at 60 to 65 °C. Errors caused by instrumental measurements are very small and within the size of markers in Figure 1. The decreasing mass fraction rate was faster for particles containing lower levoglucosan mass fractions compared to those with higher levoglucosan fractions, which is contrary to the expected behavior for ideal mixtures. This suggests that the presence of PEG accelerates the evaporation of levoglucosan due to nonidealities in the heteromolecular interactions. The particle mobility size distributions measured by SMPS prior to entering the TD are shown in Figure S3.

Levoglucosan and its isomers (mainly mannosan and galactosan) are detected by the chemical formula (C_6_H_10_O_5_) using EESI-TOF. Levoglucosan, mannosan, and galactosan have an identical carbon backbone and functional groups, and the abundance of levoglucosan is roughly over 10 times greater than that of its isomers.^21−23^ Therefore, we assume that the signal of C_6_H_10_O_5_ comes solely from levoglucosan, given the prevailing predominance of levoglucosan and its expected volatility with other dehydrated sugar isomers. As shown in Figure 1, particle-phase levoglucosan emitted from wood burning (BBPOA) evaporated faster than the particles of the pure levoglucosan standard at similar particle size and residence time in the TD. However, its evaporation slowed at higher temperatures compared to the pure standard. The slowing in evaporation at high temperatures likely comes from a diffusion limitation. Our results are direct evidence that the BBPOA matrix alters the volatility of levoglucosan both through heteromolecular interactions as well as by impacting particle viscosity and associated diffusion limitations, indicating significant nonidealities are present in BBPOA and thus the importance of taking γ into account.

### Effective Saturation Concentration and Activity Coefficient

**Figure 2:**
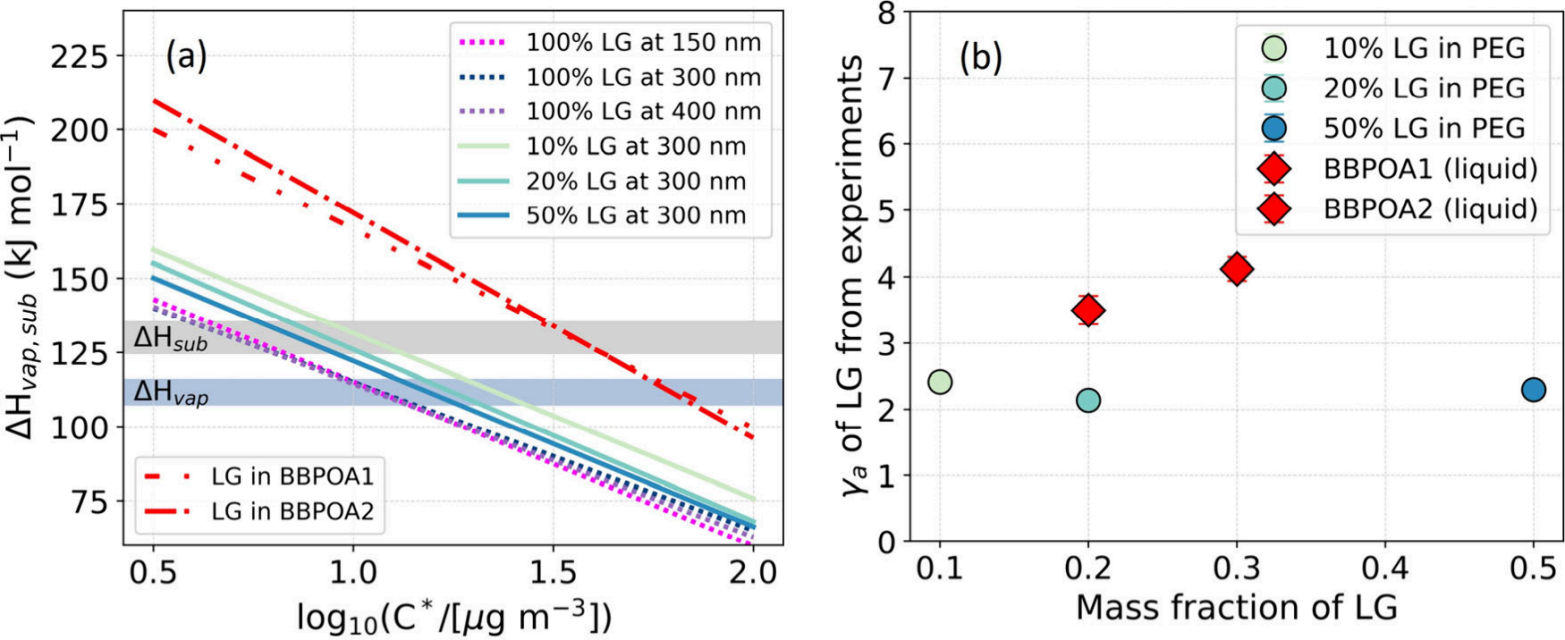
(a) The best-fit linear relationship between *ΔH* and log_10_C*of levoglucosan as determined by the volatility model. The gray and blue horizontal bands represent the enthalpies of sublimation and evaporation, respectively, of pure levoglucosan at 298 K. (b) The mole fraction-based apparent activity coefficient of levoglucosan in different matrices as determined from the experiments. The determination of the mass fraction of levoglucosan in BBPOA is described in the SI.

By inputting C*(298 K*)* ranging from 10^–3^ to 10^3^ μg m^–3^ and Δ*H* from 90 to 150 kJ mol^–1^, pairwise combinations of these parameters were iterated over in the volatility model to optimize the fit between the modeled and observed thermograms, with the fit assessed by the Pearson correlation coefficient of determination (*R*^2^). Linked by eq S3, log_10_(C*/[μg m^–3^]) and Δ*H* exhibited linearity over the studied temperature range. As shown in Figure S4, a good replication of the measurements by the model for all the experiments was obtained, as suggested by *R*^2^ > 0.99. The best-fit lines for levoglucosan in different scenarios are summarized in Figure 2a. A sensitivity test for pure levoglucosan particles at aerodynamic diameters of 150, 300, and 400 nm showed good agreement. Given that Δ*H*_*vap*_ and Δ*H*_*sub*_ do not change depending on C*, the log_10_(C*/[μg m^–3^]) of solid and liquid levoglucosan at 298 K, i.e, log_10_(*C*^0^/[μg m^–3^]), was determined, respectively, as 0.7 ± 0.2 and 1.1 ± 0.1 by finding the intercept of the best fit lines with the band of reported enthalpy of sublimation Δ*H*_*sub*_^10−12^ and vaporization Δ*H*_*vap*_.^24^ Likewise, the log_10_(C*/[μg m^–3^]) of levoglucosan with mass fractions of 10%, 20%, and 50% in the mixture of PEG was determined as 1.4, 1.3, and 1.2, respectively, using the vaporization Δ*H*_*vap*_^24^ considering that levoglucosan is dissolved in PEG and exists in the liquid state.

Particles emitted from spruce burning are likely to be liquids at 50% RH, which has been suggested in field measurements,^25^ as the high number and small mass fraction of miscible organic components with similar oxidation levels present in the BB aerosol lead to a liquid (potentially viscous) state as being thermodynamically favored.^26^ Accordingly, the intercept of the best-fit line in C* and Δ*H* space, obtained from model-measurement comparisons, with the reported Δ*H*_*vap*_^24^ was used to determine log_10_(C*/[μg m^–3^]). Therefore, the log_10_(C*/[μg m^–3^]) value of levoglucosan in spruce-burning-derived POA was estimated to be in the range of 1.8 ± 0.1. The saturation vapor concentration of pure levoglucosan at 298 K has been reported to be *C*^0^*=*13 ± 2 μg m^–3^ (log_10_(*C*^0^/[μg m^–3^])= 1.1 ± 0.07) with Δ*H*_*vap*_ of 101 kJ mol^–1^ using a thermodenuder,^8^ which is consistent with log_10_(*C*^0^/[μg m^–3^]) of 1.1 ± 0.1 reported here. The C* of levoglucosan in the ambient smoke has been reported as below 1 μg m^–3^ at 298 K, which is derived based on its evaporation at 40 to 45 °C at an altitude of 2 to 5 km.^27^ The single-temperature measurement could lead to a large uncertainty in the determination of the C*. Besides, as particles in the ambient plume age and mix with secondary aerosols, their viscosity could increase significantly due to their elevated molecular weight and oxygenated functional groups.^28−30^ The low temperature at high altitude could also promote the glass transition of aerosols.^31^ Therefore, the rise in viscosity kinetically limits gas–particle partitioning in this airborne measurement and consequently results in a lower value of C* of levoglucosan than that in fresh BBPOA.

Finally, the mole fraction-based activity coefficient γ of levoglucosan was calculated by applying eq S2 to the extracted *C*_*i*_^0^ and *C*_i_^*^ values. As shown in Figure 2b, the average γ value of levoglucosan in the mixture of PEG increased from 2.1 to 2.4 with the mass fraction of levoglucosan in the mixture ranging from 50% to 10%. The γ value larger than unity could further indicate that the system would be favorable for phase separation. The aerosol inorganic–organic mixtures functional groups activity coefficients (AIOMFAC) model, a thermodynamic group-contribution activity coefficient model, has been utilized to compare its predictions with the measurement-determined activity coefficients and to provide insights into the likely equilibrium phase state. Detailed information about the model can be found in the SI and elsewhere.^32,33^ The equilibrium AIOMFAC model predicted that at 30% RH, levoglucosan at the studied ratios in PEG mixtures would partition into two coexisting liquid phases due to a lower Gibbs energy of mixing compared to a single-phase state, as captured by the binodal coexistence curve in a phase diagram.^34^ Both levoglucosan and PEG establish the equilibrium water content and retain water at the initial 30% RH because they are hygroscopic. At equilibrium, the activities of levoglucosan would be equal in both phases and remain constant for a range of distinct initial mixing ratios that fall within the miscibility gap because the activity of water remains constant when the particles are in equilibrium with a fixed RH at room temperature. However, as the temperature increases in our experiments, the RH and particle water content decreases during evaporation, deviating from the initial equilibrium state and leading to dynamic mass transfer in the TD. For a fixed temperature, the water retained by levoglucosan evaporates proportionally alongside levoglucosan for a constant RH within the residence time in the TD, while the water retained by PEG evaporates at higher temperatures due to PEG’s lower volatility. Consequently, particles with more PEG (less levoglucosan) retain more water, which acts as a good solvent and mitigates the degree of differences in the coexisting phases during liquid-liquid phase separation (LLPS). Therefore, levoglucosan in a mixture with a high proportion of PEG exhibits a higher miscibility among the constituents over a wider temperature range. When some mixing between levoglucosan and PEG occurs in one of the phases, levoglucosan will evaporate faster due to the unfavorable interaction with PEG compared to its limited miscibility in a fully separated LLPS state, and accordingly, the activity coefficient of levoglucosan increases compared to the pure component (or the fully separated LLPS) state.

The acceleration of the evaporation rate of levoglucosan by PEG would be more obvious at lower temperatures in the TD experiment, at which point the water content is higher. This aligns with our observation that the difference in evaporation rates of levoglucosan is higher at lower temperatures while the difference becomes smaller at higher temperatures (Figure S5). The dynamic changes in water content and resulting mass transfer were not accounted for in the equilibrium scenario predicted by the model, which may explain discrepancies between the model and measurements. Therefore, the predictions of activity coefficients from the AIOMFAC model are primarily used as a qualitative indicator of the expected nonideal mixing behavior, but not used for a quantitative comparison with the measurements.

The experimentally obtained γ is based on the assumption of a well-mixed system; i.e., it represents a value for the particle overall, while the actual particle state may partially be that of a LLPS. Therefore, the measured γ serves as an apparent activity coefficient γ_*a*_, encompassing not only nonideal interactions among molecules but also a deviation introduced by potential LLPS within particles.

It is a challenge to model levoglucosan in the complex matrix of BBPOA given the thousands of unknown organic compounds in the particles. By simulating the residential wood-burning process in the laboratory, the γ_*a*_ of levoglucosan in BBPOA was experimentally determined to be around 3.8 ± 0.3, assuming the aerosols exist in a liquid-like physical state. LLPS in BB organic aerosols has been recently observed at room temperature and 50% RH.^35^ Therefore, here the reported value of γ represents either the actual or apparent (γ_*a*_) activity coefficient depending on the present phase mixing state.

The nonideal interactions between levoglucosan and PEG or BBPOA are due to the varying strength of van der Waals forces among molecules, which are weaker between levoglucosan molecules and the components in PEG or BBPOA mixtures than among levoglucosan molecules only, leading to activity coefficients of levoglucosan exceeding 1.0. The nonideal interactions can be attributed to the differences in polarity, molecular sizes, and shapes.^36^ These interactions significantly influence the partitioning of semivolatile organic compounds.^17,37^ The base-10 logarithms of the n-octanol/water partition coefficient (an indicator of polarity) for PEG-300 and levoglucosan are estimated to be −0.7 and −1.25, respectively.^38,39^ In addition, a substantial amount of POA from BB, besides levoglucosan, is less polar, and some are hydrophobic, such as hydrocarbons.^40,41^ As a result, the mole fraction-based activity coefficients of levoglucosan in PEG-300 and POA exhibit values greater than unity.

### Implications

**Figure 3:**
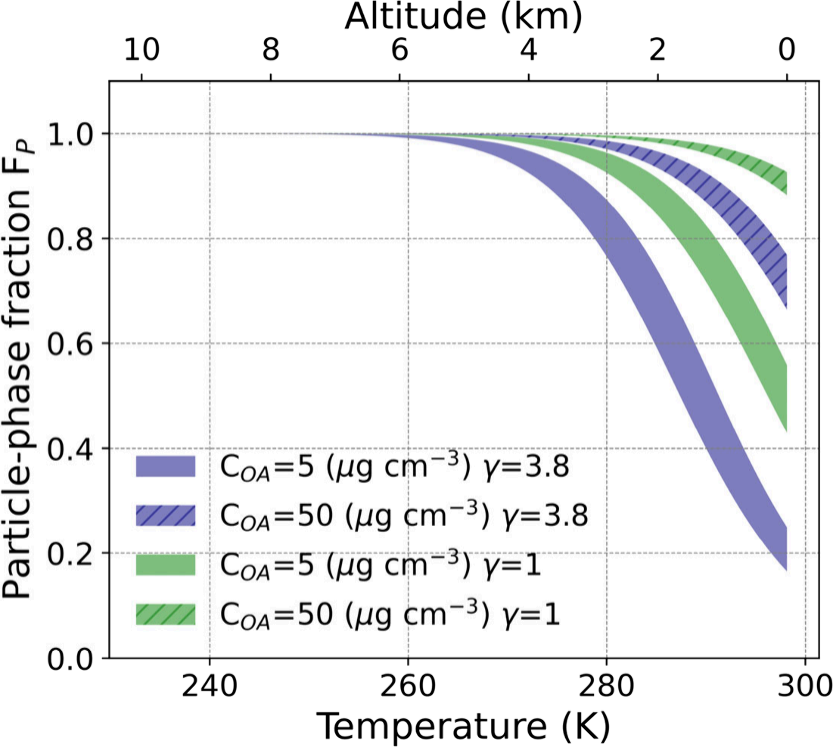
Gas–particle partitioning of levoglucosan at different temperatures (bottom axis) and the corresponding altitudes (top axis) in clean and polluted areas assuming the temperature at the surface is 298 K.

There is significant uncertainty in quantifying the BB contribution to the ambient aerosol budget using levoglucosan as a tracer due to it being semivolatile and the aforementioned matrix effects. Particularly, as we consider variations in elevation within the troposphere, temperature increases will typically lead to the loss of condensed-phase levoglucosan (also depending on the RH variation). For example, at a surface station sampling air at 298 K, one would expect the fraction of levoglucosan in the particle phase (*F*_*p*_) to be above 50% when assuming γ = 1 (under equilibrium partitioning, )^42^ when the organic aerosol loading is 5 μg m^–3^. In contrast, only ∼20% levoglucosan would be observed in the condensed phase when γ_*a*_ = 3.8 is taken into account (Figure 3). Conversely, in polluted areas with particle mass loadings of 50 μg m^–3^, if γ_*a*_ is not considered, around 90% of levoglucosan would be in the aerosol phase, while with γ_*a*_ = 3.8, around 75% of levoglucosan would be in the aerosol phase (Figure 3). Thus, consideration of γ_*a*_ creates a large difference in the expected partitioning. Neglecting the activity coefficient will lead to underestimation in assessing the BB contribution, even at high mass loadings.

In the atmosphere, the chemical composition and phase state of BBPOA undergo transformations due to oxidation reactions and changes in meteorological conditions. Consequently, the γ_*a*_ of levoglucosan is expected to change accordingly. For example, when SOA is mixed into BBPOA, the γ_*a*_ of levoglucosan might change due to different functional groups of SOA and lower levoglucosan fraction. Additionally, aged OA is likely to be viscous, thereby reducing the particle-phase molecular diffusion coefficient. The diffusion limitation also increases at higher altitudes where the temperature is lower.^31^ The evaporation of levoglucosan and associated establishment of equilibrium gas–particle partitioning of levoglucosan thus occur at a slower rate than expected. The lower value of C* of levoglucosan found by Pagonis et al.^27^ at the altitude between 2 and 5 km in the real atmosphere is likely caused by the higher viscosity of particles resulting from aged aerosol and low temperature. Consequently, unraveling the role of molecular interactions becomes challenging when they are obscured by diffusion limitations. From the perspective of practical application of our findings in global models, the effective saturation vapor concentration of levoglucosan in BBPOA reported in this paper (log_10_(C*/[μg m^–3^]) = 1.8) when it is in a liquid-like phase state) should be considered. This method can be used in future work on the quantitative gas–particle partitioning of various biomass-burning tracers and their changes with various aging pathways.

## Data Availability

The data presented in the text and figures are available in the Zenodo online repository (https://zenodo.org/records/13765208).

## Supporting Information Available

Additional experimental details and methods, including photographs of the experimental setup, TD and EESI-TOF characterization, particle diameter distribution, and model outputs (PDF)
