# Supplementary material for: Estimation of the Volatility and Apparent Activity Coefficient of Levoglucosan in Wood-Burning Organic Aerosols
Source: Environ Sci Technol Lett. 2024 Nov 3;11(11):1214–9. doi: 10.1021/acs.estlett.4c00608 (PMC11562795; doi:10.1021/acs.estlett.4c00608)
Supplement: Supplementary file 1 — ez4c00608_si_001.pdf [file ez4c00608_si_001.pdf]

Supporting Information

Estimation of the volatility and apparent activity coefficient of levoglucosan in wood-burning organic aerosols

Jun Zhang[1], Andreas Zuend[2], Jens Top[1], Mihnea Surdu[1,a], Imad EI Haddad[1], Jay G. Slowik[1], Andre S. H. Prevot[1,*], David M. Bell[1,*]

[1] PSI Center for Energy and Environmental Sciences, Paul Scherrer Institute (PSI), 5232 Villigen, Switzerland

[2] Department of Atmospheric and Oceanic Sciences, McGill University, Montréal, Quebec, H3A 0B9, Canada

[a] now at: Extreme Environments Research Laboratory, École Polytechnique Fédérale de Lausanne, 1951 Sion, Switzerland

*corresponding author; email: andre.prevot@psi.ch, david.bell@psi.ch

Supplementary Sections

The determination of effective saturation concentration and activity coefficient. The time- and temperature-dependent evaporation of species i was calculated by solving the differential equations for the measured particle-phase concentration, $C_{p,i}$, and the difference between the measured gas phase concentrations, $C_{g,i}$, and effective saturation concentration, C_i^* , of the evaporating species i, as shown below:

$$\frac{dC_{p,i}}{dt} = CS \cdot (C_{g,i} - \frac{C_{p,i}}{\sum_i C_{p,i}} C_i^*) \qquad \text{(Equation S1)}$$

Here *CS* denotes the condensation sink, which is determined by the number concentration of particles, particle diameter, and the gas-phase diffusivity. More description of the evaporation model can be found in Bell et al (in preparation). The fraction of compound i in the liquid-like mixture, $\frac{C_{p,i}}{\sum_i C_{p,i}}$, during evaporation could potentially impact the determination of $C^*$and further γ. The fraction of levoglucosan in the mixture is measured by EESI, as an input into the volatility model, effectively accounting for the mass loss of other components. For example, as components more volatile than levoglucosan evaporate, the mass fraction of levoglucosan increases commensurately (Figure S6, i.e., between 30 and 35 °C). The temperature-varying fractional abundance of levoglucosan could potentially change its thermogram by varying its partial pressure and surface area, besides the influence of non-ideal interactions. To test its sensitivity, we fixed C^* while varying $\frac{C_{p,i}}{\sum_i C_{p,i}}$ in the modeled evaporation process by considering scenarios where levoglucosan was mixed with compounds exhibiting either higher or lower volatility than levoglucosan. The result shown in Figure S7 indicates a negligible influence on the thermograms.

For a single-liquid-phase system, the mole-fraction-based activity coefficient of compound i (γ_i) can be derived from the relationship expressed by Equation S2[1]; Gorkowski et al.[2] also provide expressions for the multiphase case.

$$C_i^* = C_i^0 \gamma_i \frac{\Sigma_k C_k}{M_i \Sigma_k \frac{C_k}{M_k}} \qquad \text{(Equation S2)}$$

In Equation S2, C_i^0 denotes the gas phase saturation mass concentration of pure compound i, C_k is the mass concentration of any compound k in the system (including water), M_i and M_k are the molar masses of compounds i and k, respectively. The deviation from ideality we calculate with our approach stems either from non-ideal molecular interactions within one phase resulting in high activity coefficients or from liquid–liquid phase separation resulting in higher activity coefficient in one of the liquid phases than expected. Therefore, we will term this deviation apparent activity coefficient (γ_a) when phase separation occurs.

TD Characterization. The effective residence time through the TD heating section at room temperature was 5.2 seconds. The temperature was ramped from 20 °C to 70 °C in increments of 5 °C, held for 5 min at each temperature level. The temperature was corrected by measuring the central stream temperature in the TD (Figure S8). The particle loss caused by thermophoresis was considered; it was corrected by the measured particle number concentration at 20 °C. A bypass line at room temperature was set parallel with the TD for the correction of particle loss in the holding tank by using an exponential fit of measured mass with elapsed time.

Evaporation model. The temperature dependence of the saturation concentration C_i^*, i.e., $C_i^*(T)$, is estimated by the Clausius–Clapeyron equation with the assumption that the enthalpy of vaporization (ΔH_{vap}) or sublimation (ΔH_{sub}) is constant over the temperature range considered:

$$C_i^*(T) = C_i^*(298\,K) * \exp\left[\frac{\Delta H_i}{R}\left(\frac{1}{298\,K} - \frac{1}{T}\right)\right] \quad \text{(Equation S3)}$$

Mass fraction of levoglucosan in spruce burning POA. As shown in Figure S2, the ion flux of pure levoglucosan particles measured by EESI-TOF exhibited a good linear correlation with the mass measurements via SMPS. Consequently, it is straightforward to convert the ion flux of levoglucosan into mass concentration. The density of spruce burning POA was calculated as 1.7 ± 0.06 g m^{-3}, derived from the mobility diameter measured by SMPS and aerodynamic diameter selected by AAC, assuming the particles are spherical. By applying the density, the total volume concentration measured by SMPS was converted to the mass concentration, and thereby the mass fraction of levoglucosan in particles was determined.

AIOMFAC model and results assuming liquid-liquid phase separation (LLPS). The AIOMFAC model is a thermodynamic activity model based on the group-contribution concept of molecular interactions, with a particular interest in capturing nonideal mixing occurring in systems containing water, organic functional groups, and inorganic ions. Equilibrium models based on AIOMFAC can predict activity coefficients for compounds in multiphase systems, including vapor–liquid and liquid–liquid equilibria. A detailed description of this model and its parameterization can be found elsewhere.[3-5] Under the assumption of a well-mixed single phase, AIOMFAC predicts $\gamma_{AIOMFAC}$ values is 6.1 for 50% levoglucosan and far exceeding 10 for both 10% and 20% levoglucosan, indicating a likely liquid–liquid phase separation (LLPS) within the system.[1] Accordingly, an AIOMFAC-based equilibrium model was used that accounts for LLPS, and the result is shown in Table S1.[6] In our application, the AIOMFAC-based equilibrium model was used to predict the activity coefficients of levoglucosan in mixtures with water and PEG-5 ($C_{10}H_{22}O_6$) and PEG-6 ($C_{12}H_{26}O_7$), the most abundant compounds in PEG-300, with the same mass fractions as those used in the experiments at 30% RH and room temperature. This setup recreated the (initial) conditions of the generated aerosols in the experiments. The model predicts that the coexistence of two liquid phases results in a lower Gibbs energy for the system compared to assuming/forcing them to mix in a single liquid phase. Detailed descriptions can be found in previous studies.[6]

The effective activity coefficient ($\gamma_{eff,AIOMFAC}$) is a weighted mean activity coefficient based on the molar liquid–liquid phase partitioning of levoglucosan and the γ of levoglucosan in each phase (Equation S4). It is is calculated via weighting fractional molar abundances, $Frac_1 = \frac{n_{j,1}}{n_{j,1}+n_{j,2}}$ in phase 1, with $n_{j,1}$ and $n_{j,2}$ denoting the molar amounts of component j in phases 1 and 2, respectively (similarly, $Frac_2 = 1 - Frac_1$ for phase 2) and activity coefficients of levoglucosan (γ_1 in phase 1 and γ_2 in phase 2) in the two phases, derived from AIOMFAC model predictions, as expressed by Equation S4. This weighting is equivalent to retrieving an activity coefficient based on the activity of j (a_j) in a particle and dividing by the effective (or actual) overall mole fraction $x_{eff,j}$ in the particle phase (including in case of LLPS); since $a_j = x_{eff,j} \times \gamma_{eff,j}$ and activities of j are equal among different phases at equilibrium. This effective activity coefficient represents an appropriate mean value for the particle as a whole, such as when compared to thermodenuder experiments that treat the particle as a whole entity without information about the activity coefficients in individual phases.

$$\gamma_{eff,AIOMFAC,j} = \frac{n_{j,1}}{n_{j,1}+n_{j,2}}\gamma_{j,1} + \frac{n_{j,2}}{n_{j,1}+n_{j,2}}\gamma_{j,2} \qquad \text{Equation S4}$$

The predictions of the phase-specific activity coefficients in case of a levoglucosan-rich phase and a PEG-rich phase (see Table S1) depend on estimated interaction parameters between water, levoglucosan and PEG-300 groups in the AIOMFAC framework. The interactions between the oxyethylene groups of PEG and several groups in levoglucosan are not well constrained experimentally, making it challenging to accurately quantify uncertainties by comparing the model to real-world conditions.

Supplementary Tables

Table S1. The mole-fraction-based activity coefficient and other parameters predicted by the AIOMFAC-based equilibrium model for both a levoglucosan-rich phase (phase 1) and a PEG-rich phase (phase 2) in a mixture of water, levoglucosan, and PEG-300 at 30% RH for T = 298 K (liquid–liquid phase-separated state).

AIOMFAC-derived parameters	10% levoglucosan mixture	20% levoglucosan mixture	50% levoglucosan mixture
Fraction of levoglucosan in phase 1 to total levoglucosan (Frac1)	9.98E-01	9.99E-01	1.00E+00
Fraction of levoglucosan in phase 2 to total levoglucosan (Frac2)	2.47E-03	1.10E-03	2.74E-04
Mole fraction of levoglucosan in phase 1	0.53	0.53	0.53
Mole fraction of levoglucosan in phase 2	2.58E-04	2.58E-04	2.58E-04
Activity coefficient of levoglucosan in phase 1	0.78	0.78	0.78
Activity coefficient of levoglucosan in phase 2	1609	1609	1609
Activity of levoglucosan in the solution in phase 1	0.42	0.42	0.42
Activity of levoglucosan in the solution in phase 2	0.42	0.42	0.42
Effective activity coefficient ($\gamma_{eff,AIOMFAC}$)	4.8	2.5	1.2

Supplementary Figures

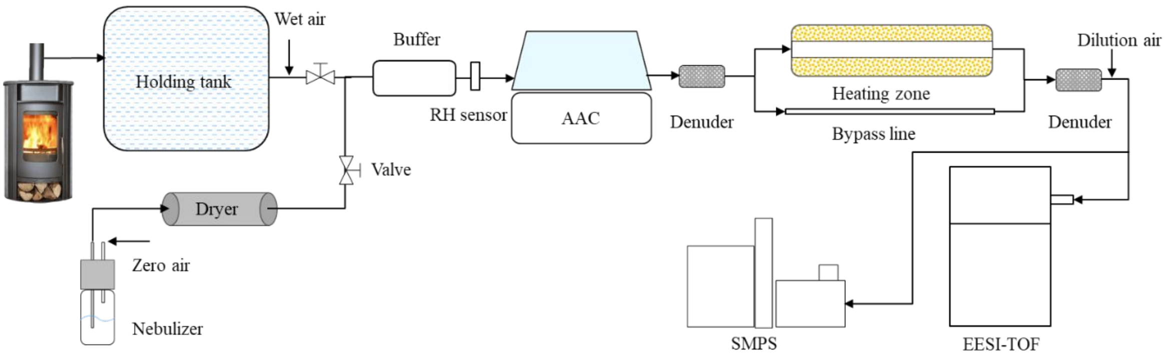

Figure S1 Experimental setup.

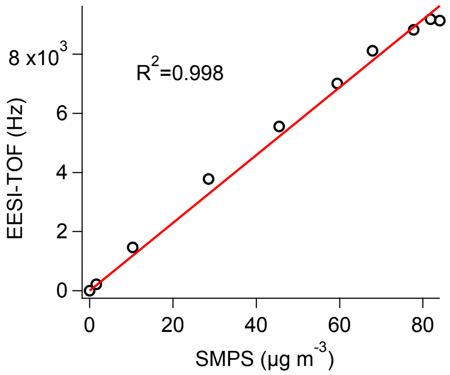

Figure S2 Ion flux of levoglucosan from EESI-TOF and the mass concentration measured by SMPS assuming spherical particles and material density of pure levoglucosan.

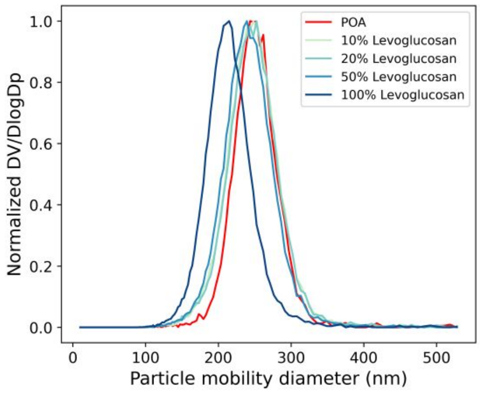

Figure S3 The mobility size distribution of particles measured by SMPS. They are all monodisperse at 300 nm aerodynamic diameter, but variations in their compositions result in differences in density and shape, leading to slightly smaller mobility diameters for pure levoglucosan (240 nm for PEG mixture vs. 212 nm for pure levoglucosan). Nonetheless, particle size does not influence the conclusions because bigger levoglucosan particles should evaporate slower because of their smaller surface-to-volume ratio, contrary to our observation.

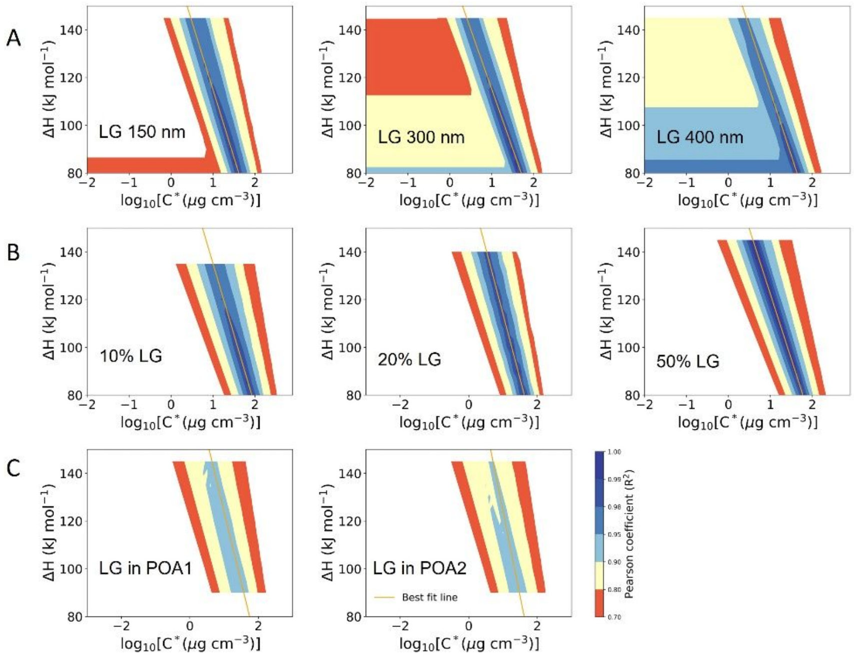

Figure S4 The potential combination of ΔH and C* and the best fitting from the volatility model. Panel (A) pure levoglucosan at different aerodynamic sizes; Panel (B) levoglucosan with different mass fractions in the mixture of PEGs; Panel (C) levoglucosan co-emitted from POA of spruce burning.

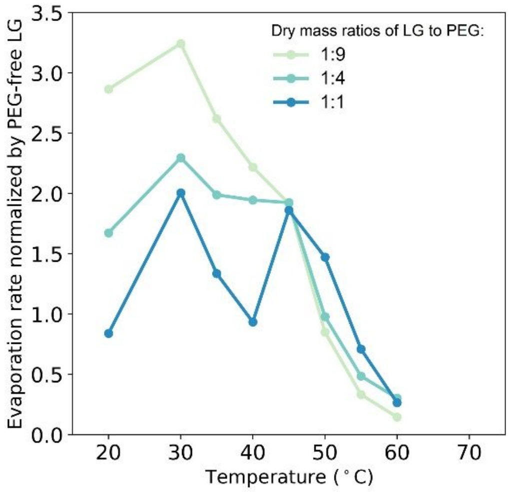

Figure S5 The evaporation rate of levoglucosan (LG) in PEG mixture normalized by the evaporation rate of PEG-free levoglucosan.

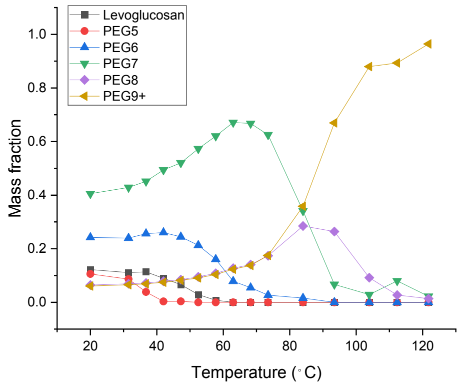

Figure S6. Mass fraction measurements of levoglucosan and PEG components for the levoglucosan:PEG dry ratio 1:9 experiment. The mass fraction of LG was then input into the evaporation model for volatility determination.

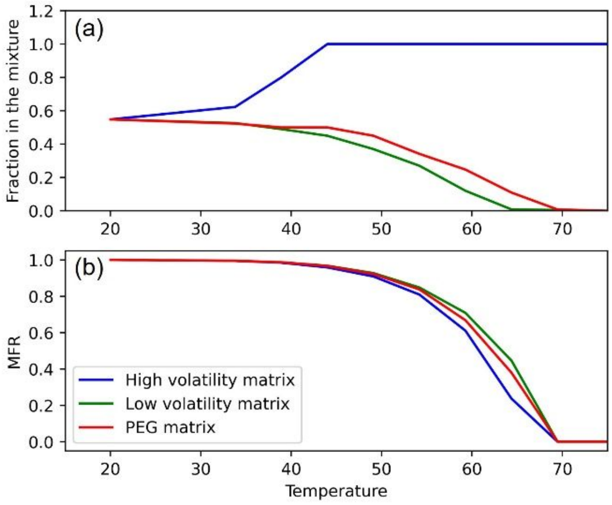

Figure S7 The sensitivity test for levoglucosan mixed in different volatility matrices. (a) The assumed mass fraction of levoglucosan in different matrices over evaporation. (b) The mass fraction remaining (MFR) of levoglucosan is calculated by the model with fixed C^* and ΔH using the fraction of levoglucosan in panel (a).

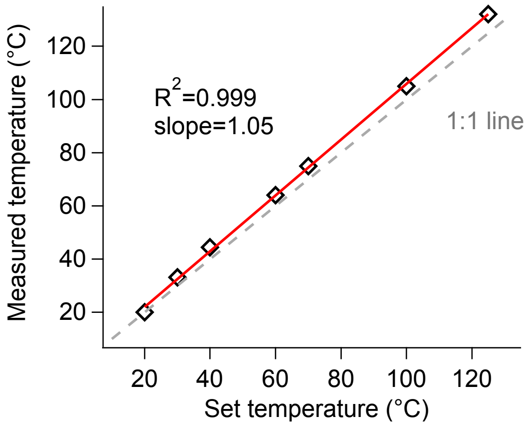

Figure S8 The set temperature and measured central line temperature in TD at a flow rate of 1 L min^{-1}.

Reference

(1) Zuend, A.; Seinfeld, J. H. Modeling the gas-particle partitioning of secondary organic aerosol: the importance of liquid-liquid phase separation. *Atmos. Chem. Phys.* **2012**, *12* (9), 3857-3882.
(2) Gorkowski, K.; Preston, T. C.; Zuend, A. Relative-humidity-dependent organic aerosol thermodynamics via an efficient reduced-complexity model. *Atmos. Chem. Phys.* **2019**, *19* (21), 13383-13407.
(3) Zuend, A.; Marcolli, C.; Booth, A. M.; Lienhard, D. M.; Soonsin, V.; Krieger, U. K.; Topping, D. O.; McFiggans, G.; Peter, T.; Seinfeld, J. H. New and extended parameterization of the thermodynamic model AIOMFAC: calculation of activity coefficients for organic-inorganic mixtures containing carboxyl, hydroxyl, carbonyl, ether, ester, alkenyl, alkyl, and aromatic functional groups. *Atmos. Chem. Phys.* **2011**, *11* (17), 9155-9206.
(4) Zuend, A.; Marcolli, C.; Peter, T.; Seinfeld, J. H. Computation of liquid-liquid equilibria and phase stabilities: implications for RH-dependent gas/particle partitioning of organic-inorganic aerosols. *Atmos. Chem. Phys.* **2010**, *10* (16), 7795-7820.

(5) Zuend, A.; Marcolli, C.; Luo, B. P.; Peter, T. A thermodynamic model of mixed organic-inorganic aerosols to predict activity coefficients. *Atmos. Chem. Phys.* **2008**, *8* (16), 4559-4593.
(6) Zuend, A.; Seinfeld, J. H. A practical method for the calculation of liquid–liquid equilibria in multicomponent organic–water–electrolyte systems using physicochemical constraints. *Fluid Phase Equilib.* **2013**, *337*, 201-213.
